# Antioxidant Effects of the Quercetin in the Jejunal Myenteric Innervation of Diabetic Rats

**DOI:** 10.3389/fmed.2017.00008

**Published:** 2017-02-07

**Authors:** Sara R. Garcia de Souza, Marcílio Hubner de Miranda Neto, Juliana Vanessa Colombo Martins Perles, Flávia Cristina Vieira Frez, Isabela Zignani, Francielle Veiga Ramalho, Catchia Hermes-Uliana, Gleison Daion Piovezana Bossolani, Jacqueline Nelisis Zanoni

**Affiliations:** ^1^Department of Morphological Sciences, Universidade Estadual de Maringá, Maringá, Paraná, Brazil; ^2^Universidade Federal de Mato Grosso do Sul, Coxim, Mato Grosso do Sul, Brazil

**Keywords:** diabetes mellitus, enteric nervous system, glia, neuroprotection, quercetin

## Abstract

**Purpose:**

Enteric glial cells (EGCs) exert a critical role in the structural integrity, defense, and metabolic function of enteric neurons. Diabetes mellitus is a chronic disease characterized by metabolic disorders and chronic autonomic neuropathy. Quercetin supplementation, which is a potent antioxidant, has been used in order to reduce the effects of diabetes-induced oxidative stress. The purpose of this research was to investigate the effects of quercetin supplementation in the drinking water at a daily dose of 40 mg on the glial cells and neurons in the jejunum of diabetic rats.

**Materials and methods:**

Twenty 90-day-old male adult Wistar rats were split into four groups: normoglycemic control (C), normoglycemic control supplemented with quercetin (Q), diabetic (D), and diabetic supplemented with quercetin (DQ). After 120 days, the jejunums were collected, and immunohistochemical technique was performed to label S-100-immunoreactive glial cells and HuC/D-immunoreactive neurons.

**Results:**

An intense neuronal and glial reduction was observed in the jejunum of diabetic rats. Quercetin displayed neuroprotective effects due to reduced cell body areas of neurons and glial cells in Q and DQ groups compared to their controls (C and D groups). Interestingly, quercetin prevented the glial and neuronal loss with a higher density for the HuC/D-immunoreactive neurons (23.06%) and for the S100-immunoreactive glial cells (14.55%) in DQ group compared to D group.

**Conclusion:**

Quercetin supplementation promoted neuroprotective effects through the reduction of neuronal and glial body areas and a slight prevention of neuronal and glial density reduction.

## Introduction

The incidence of diabetes mellitus (DM) increases every year and has been associated with the growth of obesity in the worldwide population (1, 2). The long-term presence of hyperglycemia can promote an autonomic neuropathy that affects the parasympathetic and sympathetic nerves. Furthermore, DM is associated with functional disorders of several organs, including the gastrointestinal tract. The diabetic neuropathy also affects the enteric nervous system (ENS), which promotes severe changes in the functional digestive activity (e.g., motor, secretory/absorptive, and vascular changes) (3). In particular, the ENS is a subdivision of the autonomic nervous system that comprises a neuronal network distributed throughout the digestive system, together with enteric glial cells (EGCs), which are non-neuronal cells and the major constituent of the ENS involved in the physiological activity and homeostasis maintenance of the gastrointestinal tract (4, 5).

Several researches have described that EGCs exert a structural supportive role to the enteric neurons and are also responsible for their survival and maintenance (5). The EGCs express the S100 protein (calcium-binding protein), a panglial marker that has been intensively studied and observed through the immunohistochemical techniques (6). The S100 protein exerts neurotrophic activity through the growth induction of neuronal extensions as well as dendrites and axons, and in the participation of the enteric neuronal survival. In addition, S100 protein is also involved in the regulation of specific intracellular signaling pathways, such as the regulation of protein phosphorylation, enzymatic activities, Ca^2+^ homeostasis, cytoskeletal stability, and apoptosis induction (7). The EGCs are also involved in the metabolic support of the SNE, enteric neurotransmission, motility, and bowel inflammatory diseases (8, 9).

Neurodegeneration is characterized by the damage on the structure, function, and number of neurons, which results in essential functional deficiencies (10). The reduction of the enteric neuronal and glial density (11–13) has been well established in the diabetic neuropathy due to an increased oxidative stress, which leads to an imbalance between the production of reactive oxygen species (ROS) and the cellular antioxidant defense system (14). Furthermore, morphometric analyses of the neuronal and glial body area have also been studied for a better evaluation of the diabetic neuropathy and its neurological consequences.

Therapeutic approaches have demonstrated that the diet supplementation with antioxidants reduces the oxidative stress or inhibits the aldose reductase, an enzyme that displays a crucial role in the treatment of diabetes and its neurological complications (15). Flavonoids are a group of polyphenols that exert a remarkable antioxidant activity, and they have been substantially used for the treatment of several diseases (16). Quercetin is one of the major natural polyphenolic flavonoids, which exerts beneficial pharmacological effects, such as anti-hypertensive, antidepressant, anti-arrhythmic, anti-hyperalgesic, hypocholesterolemic, anti-hepatotoxic, anticarcinogenic, anti-ulcer, antiviral, antithrombotic, anti-ischemic, anti-inflammatory, and anti-allergy effects (17–20).

Quercetin neuroprotective effects have been described in some neurodegenerative diseases such as Alzheimer’s disease, Parkinson’s disease, and Huntington’s disease (10). Furthermore, it has recently been reported that quercetin demonstrated neuroprotective effects in the cecum and duodenum in diabetes experimental models (21, 22). Thus, the purpose of this investigation was to evaluate the quercetin supplementation effects during 120 days in the drinking water at a dose of 40 mg/day on the general neuronal population and the glial cells of jejunal myenteric plexus of diabetic rats.

## Materials and Methods

### Animals

All procedures described in the current study are in accordance with the ethical principles adopted by the SBCAL (Brazilian Society of Sciences Laboratory Animal) and have previously been submitted to analysis by the Ethics Committee on Animal Experiments of the State University of Maringá (UEM) (acceptance 053/2009).

Twenty 90-day-old male adult Wistar rats (*Rattus norvegicus*) from Central Viverium (UEM) were used. The experiment of DM contained four groups with five rats per group: normoglycemic control (C), normoglycemic control supplemented with quercetin (Q), diabetic (D), and diabetic supplemented with quercetin (DQ).

Animals were maintained in individual cages for a period of 120 days in a vivarium with a 12-h light: dark cycle and room temperature (24 ± 2°C), receiving food and water *ad libitum*. All groups received balanced standard Nuvital feed (Nuvilab, Colombo, Parana, Brazil). For the experimental supplementation of Q and DQ groups, water supplemented with quercetin (Cromofarma, São Paulo, Brazil) was given at a dose of 40 mg/day solubilized in sodium hydroxide (NaOH) at pH 7.4. The animals of C and D groups, the non-supplemented groups, only received the vehicle that contained water diluted in NaOH.

For calculation of the quercetin dose, a preliminary evaluation of water intake of Q and DQ groups was performed during three consecutive days in order to obtain the average amount of water consumed per animal.

The diabetes experiment was induced after a 14-h fasting through the intravenous injection of streptozotocin (35 mg/kg body weight; Sigma, St. Louis, MO, USA) dissolved in a citrate buffer solution at a pH 4.5 (10 mM). After 4 days of DM induction, blood glucose concentrations were measured by using the glucose oxidase method to confirm the establishment of the experimental model (23). Only the animals with blood glucose concentrations above 250 mg/dL were kept in D and DQ groups.

### Collection and Processing of the Material

After a period of 120 days, the animals were weighed and euthanized under anesthesia with thiopental (40 mg/kg body weight, i.p.; Abbott Laboratories, Chicago, IL, USA). The blood was collected by using cardiac puncture for the blood glucose measurements (20). After celiotomy, the samples were collected from all the animals, and then washed with phosphate-buffered saline (PBS, 0.1M, pH 7.4), gently inflated with Zamboni’s fixative solution to fill the space previously occupied by the stool, thus avoiding the jejunal stretching. After that, the jejunums were maintained for 18 h in the same solution at 4°C. After fixation, the samples were carefully opened along the mesenteric border and successively rinsed with 80% ethanol in order to completely remove the fixative excess. Then, the dehydrations were sequentially performed with ethanol concentration of 95 and 100%, followed by clarification in xylene. After that, the rehydration was performed by using ethanol at decreasing concentrations (100, 90, 80, and 50%). The tissues were stored at 4°C in PBS with 0.08% sodium azide.

The samples were microdissected under a stereomicroscope in order to obtain the whole mounts of muscularis tunica by the removal of the mucosal and submucosal layers. The tissues were double stained by immunohistochemistry to label HuC/D and S-100 proteins (24, 25).

### Double Staining Immunohistochemistry for the HuC/D and S100 Proteins

Twenty whole mounts were initially rinsed twice in PBS solution that contained 0.5% Triton X-100 (Sigma) for 10 min. After that, the tissues were incubated for 1 h with a blocking solution that contained 0.5% Triton X-100, 2% bovine serum albumin (BSA) (Sigma) with 10% goat serum in PBS solution. After blockage, the samples were incubated for 48 h at 4°C with a solution that contained specific primary antibodies for HuC/D (produced in mice, 1:500; Molecular Probes, Carlsbad, CA, USA) and S100 (produced in rabbits, 1:200; Sigma). The jejunums were rinsed twice in PBS solution that contained 0.5% Triton X-100 for 10 min and incubated for 2 h at room temperature with the following secondary antibodies: Alexa Fluor 488 IgG anti-mouse produced in donkey (1:500; Molecular Probes) for labeling the HuC/D protein, and Alexa Fluor 546 IgG anti-rabbit produced in goat (1:500; Peninsula Labs, Torrance, CA, USA) for labeling the S100 protein. After incubation, the whole mounts were also rinsed twice in PBS solution, mounted on slides with antifade medium, and stored in refrigeration at 4°C. For the negative control, the immunolabeling was performed without presence of primary antibody.

### Immunohistochemistry for Quantitative Analysis

To quantify the HuC/D-immunoreactive myenteric neurons (HuC/D-IR) and the S100-immunoreactive glial cells (S100-IR), the images were obtained and randomly counted and measured in the intermediate region of the jejunum. For that, the bowel circumference was divided into three regions: mesenteric, intermediate, and antimesenteric (26).

Images were captured by using the high-resolution camera AxioCam (Zeiss, Jena, Germany) coupled to a light microscope Axioskop Plus (Zeiss), and digitalized into a computer with the AxioVision Software version 4.1. The image analysis of the Image-Pro Plus version 4.5.0.29 Software (Media Cybernetics, Silver Spring, MD, USA) was used for the neuronal and glial quantification.

For each animal, all neurons and EGCs were counted in the 32 images captured with 20× objective lens. In addition, the myenteric glial cells and neurons observed inside and outside of the myenteric ganglion were considered as intraganglionar and extraganglionar, respectively. The sum of all the neurons observed in the field (intraganglionar and extraganglionar) comprised the total number of neurons. The area of each image was measured by using the Image-Pro Plus Software, which was approximately of 0.23 mm^2^. Neuronal and glial densities were expressed as the number of neurons and glial cells per square centimeter.

### Immunohistochemistry for Morphometric Analysis

Cell body areas of HuC/D-IR neurons and S100-IR glial cells were measured with the same images used for the quantitative analysis. The areas (square micrometer) of neuronal and glial body areas were measured by using an Image-Pro Plus Software, with a total of 100 cell body areas for the neurons and glial cells measured per animal.

### Statistical Analysis

Data were statistically analyzed by using the Statistica 7.1 and GraphPad Prism 5.1 Software and were expressed as mean ± SE. Morphometric data were set in delineation blocks, followed and analyzed by the Tukey’s test. For the other data, the one-way analysis of variance (ANOVA) was performed, followed by the Tukey’s test. The *p* values <0.05 were considered statistically significant.

## Results

### Physiological Data

After 120 days of diabetes induction, the animals of D (564.4 ± 45.33 mg/dL) and DQ groups (542.4 ± 46.18 mg/dL) exhibited an increased hyperglycemia compared to the C group (149.1 ± 25.32 mg/dL) (*p* < 0.001). However, quercetin supplementation promoted unchanged blood glucose concentrations in Q group (144.7 ± 16.33 mg/dL) (*p* > 0.05). Furthermore, the body weight of normoglycemic animals was 521.1 ± 15.07 g (C group) and 500.0 ± 12.46 g (Q group) (*p* > 0.05). However, the diabetic rats of D and DQ groups exhibited a reduced body weight of 331.0 ± 16.39 and 321.7 ± 20.25 g, respectively (*p* < 0.05).

### Quantitative Analysis

In the myenteric plexus, D group exhibited a density reduction of HuC/D-IR neurons (intraganglionar, extraganglionar, and total) compared to the C group (Table 1; *p* < 0.05). In addition, the myenteric neuronal density seen in DQ group demonstrated a non-significant statistical difference compared to the D group (*p* > 0.05).

**Table 1:** **Density per unit area (cm^2^) of HuC/D-immunoreactive myenteric neurons (intraganglionar, extraganglionar, and total) in the jejunum obtained from the following groups: normoglycemic control (C), normoglycemic control supplemented with quercetin (Q), diabetic (D), and diabetic supplemented with quercetin (DQ)**.

	Intraganglionar	Extraganglionar	Total	S100/Hu
C	26,646.7 ± 1,341.9	198.4 ± 9.2	26,845.1 ± 1,343.5	2.33 ± 0.05
Q	22,491.8 ± 1,610.8	176.6 ± 25.8	23,008.2 ± 1,851.7	2.37 ± 0.09
D	16,741.8 ± 739.6**	163.0 ± 20.2**	16,904.9 ± 750.0**	2.37 ± 0.02
DQ	20,633.2 ± 873.3	171.2 ± 14.6	20,804.3 ± 876.9	2.22 ± 0.06

*Results were expressed as mean ± SE*.

*n = 5 rats per group*.

**Significant difference between D vs C groups according to Tukey’s test (*p* < 0.05).

There were no significant differences in the S100/Hu ratio when compared with all the groups (*p* > 0.05; Table 1).

Density of S100-IR enteric glial cells (intraganglionar, extraganglionar, and total) was significantly reduced in D group compared to the C group (Table 2; *p* > 0.05). In contrast, non-significant differences were seen between D and DQ groups (*p* < 0.05).

**Table 2:** **Density per unit area (cm^2^) of S100-immunoreactive myenteric glial cells (intraganglionar, extraganglionar, and total) in the jejunum obtained from the following groups: normoglycemic control (C), normoglycemic control supplemented with quercetin (Q), diabetic (D), and diabetic supplemented with quercetin (DQ)**.

	Intraganglionar	Extraganglionar	Total
C	52,260.9 ± 3,257.4	10,214.7 ± 821.4	62,475.5 ± 3,305.0
Q	45,005.4 ± 2,248.7	8,864.1 ± 735.1	53,885.9 ± 2,811.9
D	32,807.1 ± 1,418.6**	7,353.3 ± 703.6**	40,160.3 ± 1,892.5**
DQ	39,141.3 ± 1,223.6	6,861.4 ± 179.3	46,002.7 ± 1,227.6

*Results were expressed as mean ± SE*.

*n = 5 rats per group*.

**Significant difference between D vs C groups according to Tukey’s test (*p* < 0.05).

### Cell Body Area of the HuC/D-IR Myenteric Neurons

**Figure 1:**
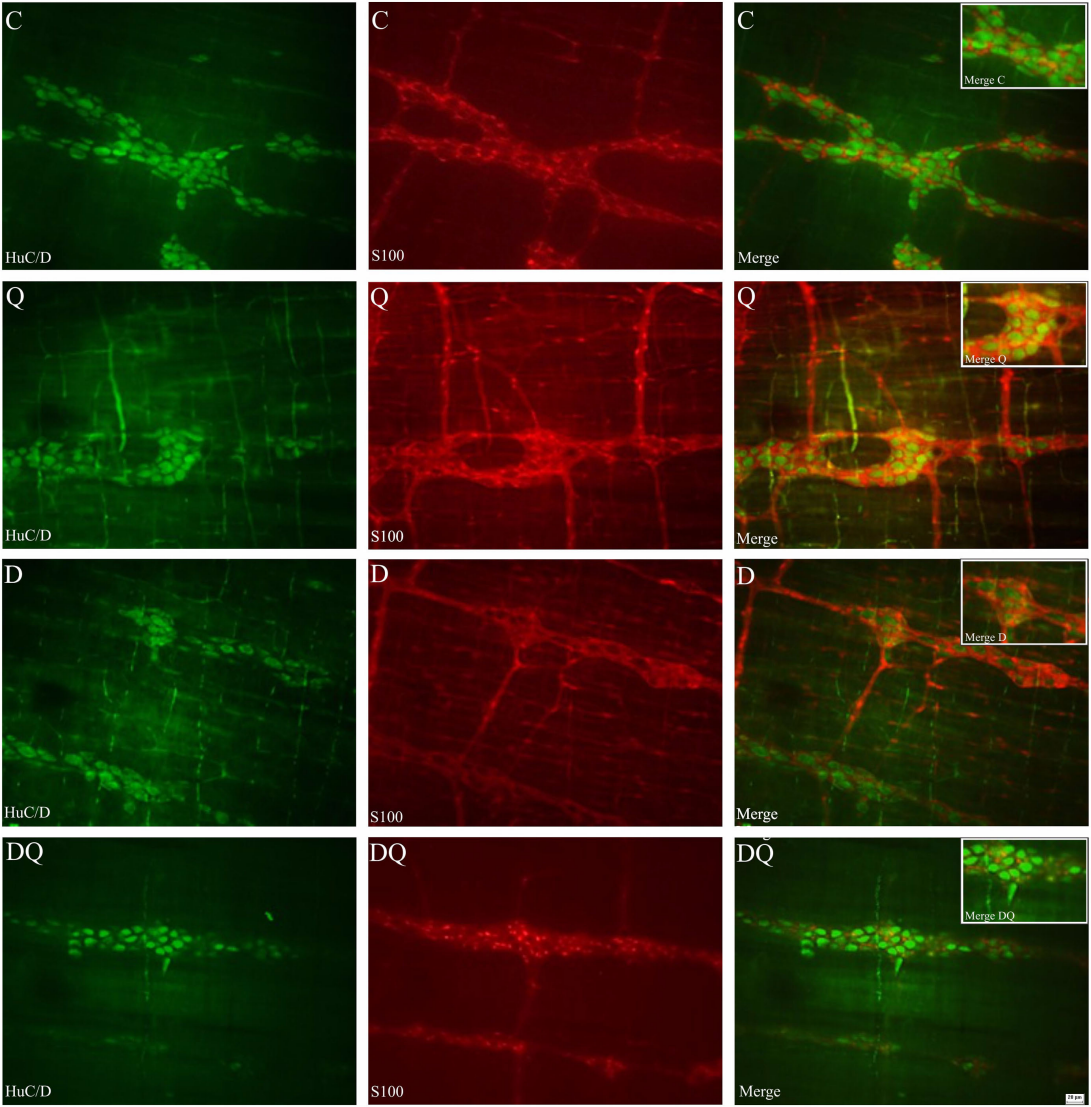
**Representative photomicrographs that illustrate the whole mounts of jejunal myenteric plexus stained by immunohistochemical technique for HuC/D-immunoreactive neurons (first column) and S-100- immunoreactive glial cells (middle column). The third column illustrates the overlapping of the images of the neurons and enteric glial cells**. The following groups were used: normoglycemic control (C), normoglycemic control supplemented with quercetin (Q), diabetic (D), and diabetic supplemented with quercetin (DQ). Scale bar = 20 µm.

Representative photomicrographs of HuC/D-IR myenteric neurons and myenteric glial cells are shown in Figure 1.

**Figure 2:**
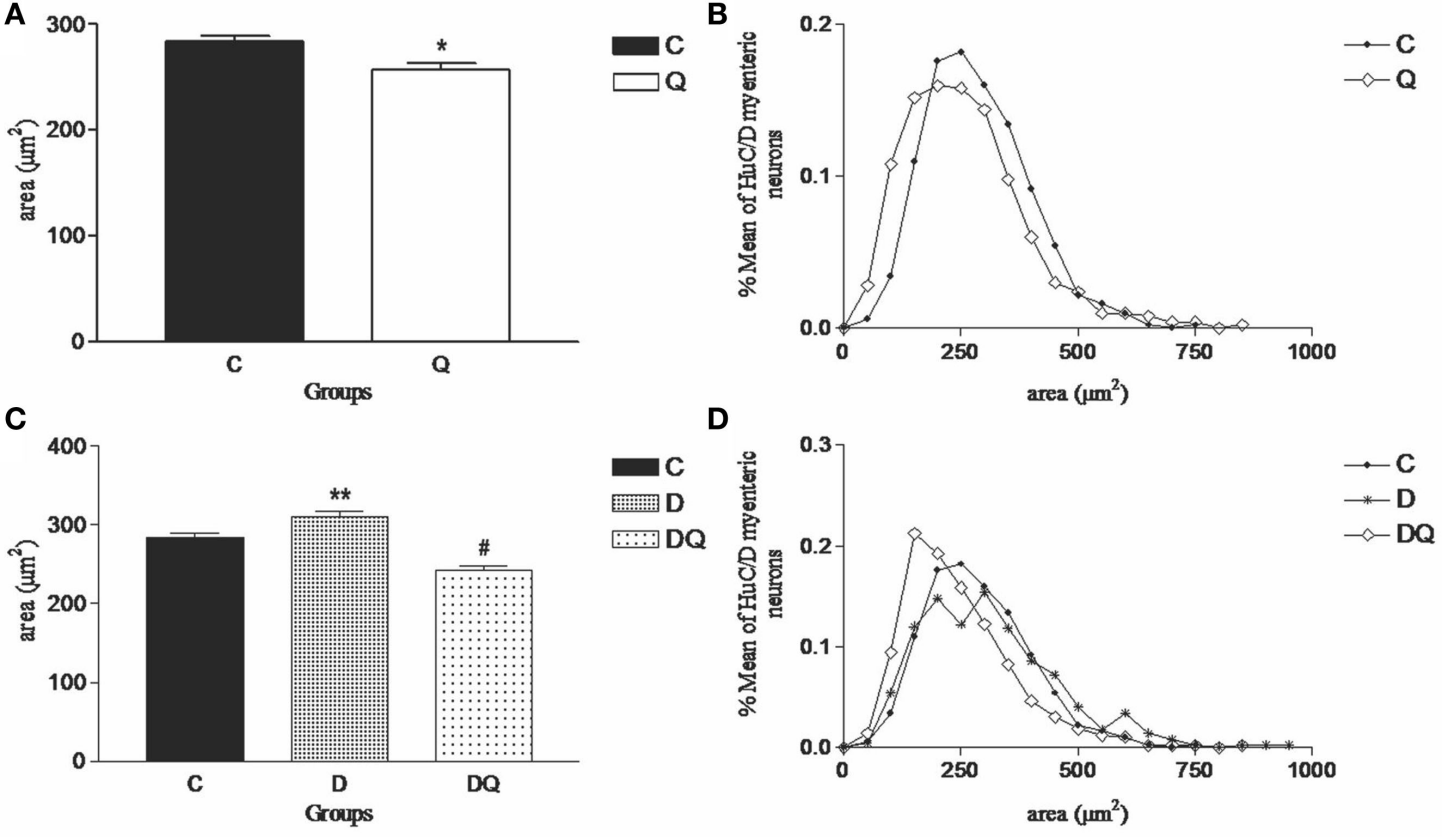
**Morphometric results of HuC/D-immunoreactive myenteric neurons**. The left column demonstrates **(A,C)** the mean ± SE of the neuronal body areas (square micrometer) and the right column illustrates the relative frequency distribution **(B,D)** of the neuronal body areas in the following groups: normoglycemic control (C), normoglycemic control supplemented with quercetin (Q), diabetic, and (D) diabetic supplemented with quercetin (DQ). *n* = 5 mice per group. *p < 0.05 vs C group; **p < 0.05 vs C group; ^#^*p* < 0.05 vs D group.

Quercetin supplementation promoted a decreased neuronal body area of HuC/D-IR myenteric neurons (*p* < 0.007) in Q group compared to the C group by observing the displacement of the distribution curve to the left (Figures 2A,B). Unlike Q group, the diabetes (D group) induced an increased neuronal body area for the HuC/D-IR neurons (*p* < 0.03) (Figure 2) in relation to the C group. Furthermore, quercetin supplementation in the diabetic animals (DQ group) restored the neuronal body area similar to those observed in the control (C group; *p* > 0.05) (Figure 2C). This result was evident on observing the displacement of the distribution curve to the left when the DQ group was compared to D (Figure 2D).

### Cell Body Area of the S100-IR Myenteric Glial Cells

**Figure 3:**
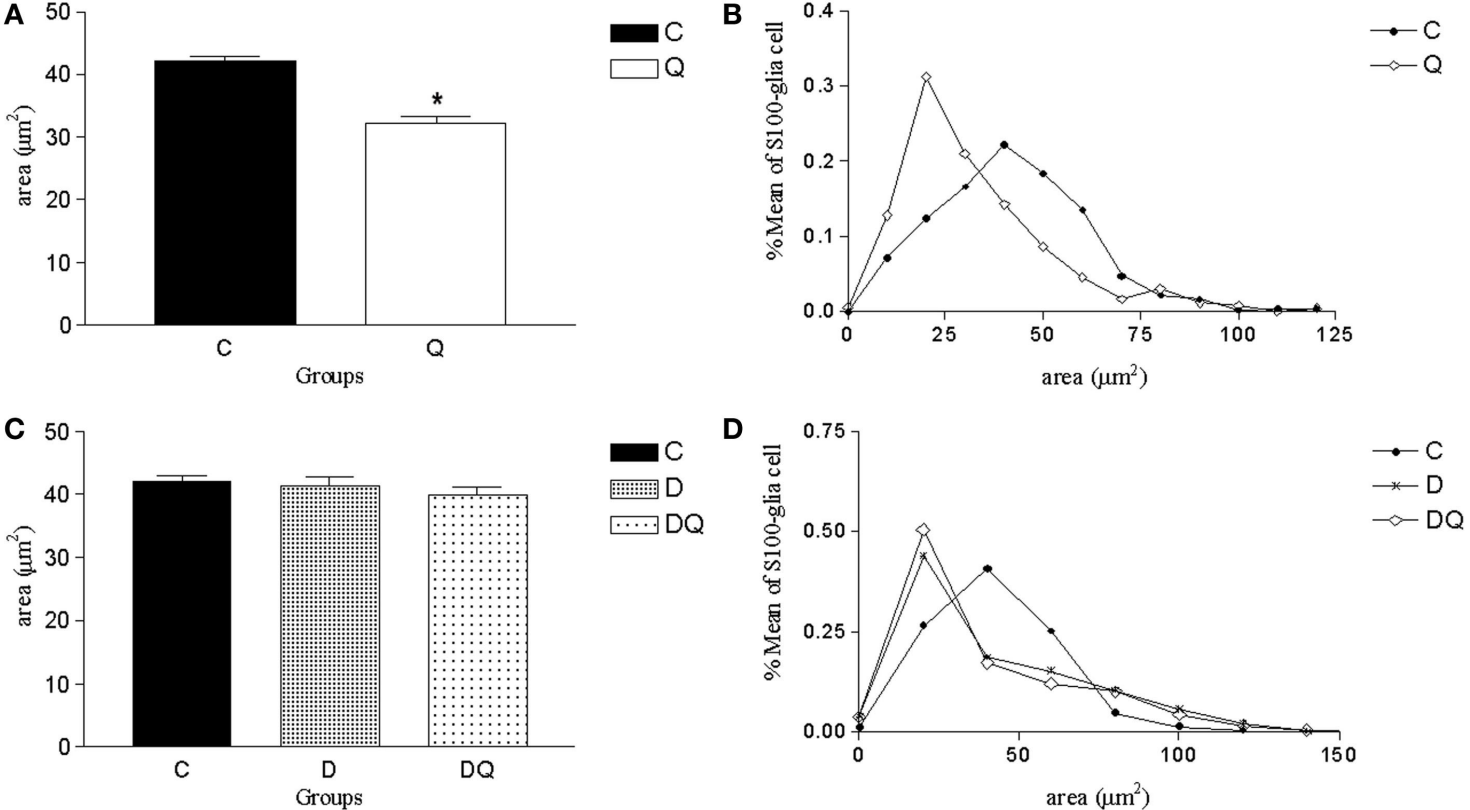
**Morphometric results of S100-immunoreactive glial cells of the myenteric plexus**. The left column shows **(A,C)** the mean ± SE of the areas of the enteric glial cells (square micrometer), and the right column illustrates the relative frequency distribution **(B,D)** of glial body areas in the following groups: normoglycemic control (C), normoglycemic control supplemented with quercetin (Q), diabetic (D), and diabetic supplemented with quercetin (DQ). *n* = 5 mice per group. *p < 0.05 vs C group.

The glial body area was lower in Q group compared to the C group (*p* < 0.05; Figure 3). However, C, D, and DQ groups demonstrated similar data in the glial body area (*p* > 0.05; Figure 3).

## Discussion

The experimental model of diabetes was confirmed by the high blood glucose concentrations observed in D and DQ groups. In addition, remarkable clinical features of DM were also seen throughout the experiment, such as weight loss, overeating, polydipsia, polyuria, hyperglycemia, and increased irritability after 7 days of DM induction, as reported by Furlan et al. (27). The body weight loss in the diabetic animals (D group) suggests a disorder in the synthesis and storage of energy reserves (28). However, quercetin did not prevent the body weight loss in the rats of DQ group compared to the D group as well as the plasma glucose levels remained unaltered. Thus, high blood glucose levels persisted throughout the experiment and its consumption was reduced in insulin-dependent tissues, thereby it might be suggested that quercetin exerts no interference in the signaling pathways that lead to a weight gain or other signaling pathways associated with the mobilization of energetic reserves in the diabetic animals (22).

Reduction of neuronal and glial density was observed in diabetic animals; similar results have been described in the literature (29–34). Enteric neuropathy is related to intracellular signaling disorders associated with quantitative and neurochemical alterations of the enteric neurons. These changes may explain the neuronal density loss and the important clinical dysfunctions of neurological alterations of DM (35). Furthermore, it has been proposed that the induction of oxidative stress might be a determinant of the neuronal damage in the diabetes as well as alterations in the cellular metabolic pathways, which may result in programed cell death through the apoptosis and autophagy (36–40).

In this study, the density data are reinforced by the results obtained in the morphometric analysis, since the diabetic rats (D group) exhibited the highest neuronal body areas. The hypertrophy of the neuronal size of diabetic rats might be associated with an increased enzymatic synthesis and as a compensatory mechanism against the neuronal density reduction. An increased functional neuronal activity likely occurs in order to balance the neuronal density loss and maintain the functional intestinal activity closer to the ideal. This feature may explain the increase of neurotransmitter production due to a compensatory effect of neuronal loss in DM (41–43). Furthermore, it has also been suggested that a defective axonal transport of neuronal proteins that are synthesized inside the neuronal body can explain the accumulation of these proteins (44). Other approach is the accumulation of oxidative material inside the cell that results in swelling of the enteric neurons (45).

In D group, the glial density was significantly lower than C group, although the glia/neuron ratio remained unchanged. In addition, diabetes-induced neuronal loss was accompanied by a proportional reduction of the glial cells. According to Burnstock, the glia/neuron ratio appears in a 2:1 relation and has been maintained in different experimental groups (46). Other essential roles of EGCs have been described such as detoxification of metabolites produced from the degradation of neurotransmitters and glutamate of extracellular medium as well as the EGCs, which are involved in the glutathione production for its consumption and exportation to the neurons, and in the participation in inflammatory processes that affect the nervous tissues (47, 48).

Although the glial density reduction has occurred, the glial body area was maintained. Few researches in the literature have analyzed the size of the glial cells in conditions of diseases and inflammatory processes. Our group’s research has already observed that diabetes leads to size reduction of EGCs (49), although hypertrophy occurs in the enteric neurons. Furthermore, EGCs provide mechanical structural support to the neurons, in addition to glial cells can also release numerous neurotrophic factors that control their development, survival, and differentiation, and these cells release reduced glutathione or glial cell-derived neurotrophic factor (GDNF) that activates the neuropeptide Y and antioxidant system, thus protecting against the neuronal death (5, 50, 51). Therefore, the EGCs loss observed in D group might be closely associated with the neuronal degeneration and gastrointestinal motility dysfunctions (51, 52).

Therapy with antioxidants has intensively been studied to prevent the enteric neuropathy progress by acting in the metabolic pathways that generate oxidative stress and/or directly on the scavenging of ROS. Quercetin, a bioflavonoid antioxidant, has been described as a chelating agent of metals and free radical scavenger (53). Furthermore, quercetin neuroprotective effects have also been studied in the diabetes and its complications induced by the oxidative stress (21, 22).

The comparison of the density of the HuC/D-IR myenteric neurons and S100-IR glial cells between the DQ and D groups has not revealed a significant statistical difference. However, the supplementation with quercetin showed a preservation of the myenteric neurons, since a total neuronal and glial density of 23.06 and 14.55% was higher in the DQ group than D group, respectively. Although quercetin slightly prevents the loss of enteric neurons, this bioflavonoid promotes a preservation of the neuronal density in most animals when analyzed individually (data not shown).

Neuroprotection effects were observed in the neuronal body size in the DQ group. The quercetin protective results were obtained in DQ group possibly due to scavenging action mechanisms underlying the neutralization of ROS and, in turn, prevention of oxidative stress-induced neuronal damage, thereby acting on the preservation of cellular enzymatic machinery (21, 22).

With regard to the neuronal and glial densities between C and Q groups, similar data were seen. For this reason, these results suggest that the daily dosage of 40 mg quercetin displayed non-neurotoxic effects due to the absence of reduction of neuronal or glial cell density in Q group. In contrast, neuroplasticity mechanisms were observed due to reduction of the myenteric neuronal and glial body area in Q group compared to C group. These results can be observed through the displacement of the frequency distribution curves to the left in Figures 2B,D and 3B,D. The smaller neuronal body area of 9.31% in Q group when compared to C group suggests that quercetin reduces the cellular disorders during lifetime such as aging and inflammatory processes that would lead to an increased cell body area and cellular physiology complications (54–56). Likewise, the smaller glial body area of 23.10% in Q group in relation to its control (C group) also suggests quercetin beneficial effects for the EGCs. It is important to highlight that EGCs and enteric neurons form an intense integrated system and a reciprocal beneficial effects may occur, thus quercetin may stimulate the concomitant survival of EGCs and neurons.

In conclusion, drinking water supplemented with quercetin at a daily dose of 40 mg displayed non-neurotoxic effects on EGCs and myenteric neurons of normoglycemic rats. Diabetes promoted an intense reduction of neurons and glial cells in the jejunal myenteric plexus of diabetic rats. Interestingly, quercetin supplementation slightly attenuated the loss of neuronal and glial cell density. In addition, neuroprotective effects were demonstrated by the presence of reduced neuronal and glial body areas in Q and DQ group, which suggests an antioxidant capacity of quercetin that prevents cellular disorders associated with long-term consequences of diabetes.

## Author Contributions

SR, MN, JP, and JZ designed the study. SR, JP, FF, IZ, and FR performed the experiments. SR, JP, CH-U, and JZ analyzed the data. SR, MN, JP, CH-U, GB, and JZ wrote the manuscript. All the authors approved the final version of the manuscript.

## Conflict of Interest Statement

The authors declare that the study was conducted in the absence of commercial or financial relationships that could create potential conflicts of interest.
